# Stability of Transgene Inheritance in Progeny of Field-Grown Pear Trees over a 7-Year Period

**DOI:** 10.3390/plants11020151

**Published:** 2022-01-06

**Authors:** Vadim Lebedev

**Affiliations:** Branch of the Shemyakin-Ovchinnikov Institute of Bioorganic Chemistry, Russian Academy of Sciences, Prospekt Nauki 6, 142290 Pushchino, Moscow Region, Russia; vglebedev@mail.ru

**Keywords:** GUS activity, Mendelian segregation, *Pyrus communis*, seed quality, tree breeding, unintended effects

## Abstract

Breeding woody plants is a very time-consuming process, and genetic engineering tools have been used to shorten the juvenile phase. In addition, transgenic trees for commercial cultivation can also be used in classical breeding, but the segregation of transgenes in the progeny of perennial plants, as well as the possible appearance of unintended changes, have been poorly investigated. We studied the inheritance of the *uidA* gene in the progeny of field-grown transgenic pear trees for 7 years and the physical and physiological parameters of transgenic seeds. A total of 13 transgenic lines were analyzed, and the *uidA* gene segregated 1:1 in the progeny of 9 lines and 3:1 in the progeny of 4 lines, which is consistent with Mendelian inheritance for one and two transgene loci, respectively. Rare and random deviations from the Mendelian ratio were observed only for lines with one locus. Transgenic seeds’ mass, size, and shape varied slightly, despite significant fluctuations in weather conditions during cultivation. Expression of the *uidA* gene in the progeny was stable. Our study showed that the transgene inheritance in the progeny of pear trees under field conditions occurs according to Mendelian ratio, does not depend on the environment, and the seed vigor of transgenic seeds does not change.

## 1. Introduction

Genetic engineering is a powerful tool for creating new genotypes of woody plants with valuable traits. Firstly, genetic engineering makes it possible to transfer genes between very divergent organisms [1], while classical breeding allows crosses only between closely related species. Secondly, it significantly shortens the time necessary for developing a new genotype; in classical breeding, this time depends on the length of the juvenile period. The latter is quite long in fruit trees: e.g., apples start to flower at the age of 4 to 10 years or more, depending on the cultivar [2]. Thirdly, genetic engineering can be used to transfer the gene of the desired trait without concurrent transfer of unwanted linked alleles, whose removal would otherwise require a long time. It takes at least 15–20 years to obtain a new apple cultivar by crossing known ones and at least 50 years to confer a wild apple trait to a new cultivar because of the need for several breeding cycles to get rid of the associated undesirable alleles [3].

The important task of shortening the breeding process in woody plants is solved by reducing the juvenile phase using a special direction of genetic engineering. By transferring specific genes to fruit [4,5,6,7] and forest [8,9,10] trees, researchers achieved their flowering at the age of several months. Based on these plants, rapid-cycling breeding systems (fast-track) were developed for apple [11], plum [12], and citrus [13], allowing dramatically shortened generation time. In addition, commercially grown transgenic trees can be used as donors of new traits in classical breeding. Finally, a shorter juvenile period may be an unintended effect of transformation by other genes, e.g., the glutamine synthetase gene in birch [14]. It is necessary to know how the transgenes will segregate in the generative progeny in all cases.

The expression stability and inheritance rules of transgenes are a major prerequisite for the application of transgenic plants [15,16]. Transgene inheritance was investigated mainly in annual crops. According to Mendelian fashion, transgenes are usually inherited in the progeny as a dominant trait, with segregation [17]. For instance, rice progeny produced by self-pollination had a segregation ratio of 3:1, 15:1, 63:1, or 255:1 in the case of 1, 2, 3, or 4 transgene copies, respectively [18]. Segregation can be stable and persist over several generations [19]. At the same time, the non-Mendelian inheritance is well-known occurred at a frequency from 10% to 50% of the analyzed lines [20]. Several hypotheses have been proposed to explain this, including the poor transmission of transgenes, T0 chimerism, gametic lethality, homozygous, multiple independently assorting insertion loci, and transgene silencing induced by multiple transgene copies, DNA rearrangements [21,22].

Because of the need for very time-consuming studies, much less information is available about transgene segregation in the progeny of woody plants. The first transgenic apple trees took 5 years to produce flowers [23]. While inheritance in annual plants was studied up to the ninth generation [24], studies in trees mostly report about the first generation, except for studies in trees with early-flowering genes [13,25]. Most studies were done in a greenhouse, and very few were in the field [16,26,27]. Unlike crops, the same tree can be used as a male/female parent for many years. Therefore, the stability of over-time rather than transgenerational inheritance comes to the fore in woody plants. Over time stability of inheritance may be influenced by changing environment, but relevant studies are very rare. Besides, transgenic plants may show unintended changes unrelated to the nature of the transferred gene, which can be caused by gene insertion, random mutations during the transformation and cultivation in vitro, or pleiotropic effects of new protein, and they cannot be predicted in advance [28]. Such unintended effects can be detected by comparing transgenic genotypes with the original ones, usually in terms of productivity, pest and disease resistance, and other important traits. Little attention is given to other traits, e.g., seed vigor, responsible for fast and uniform germination. It is a complex seed property involving regulatory networks that integrate genetic programs, metabolic signals, and hormonal signaling pathways [29]. Unintended effects can disrupt the processes of seed development on the mother plant and thus affect the seed vigor. This trait is considered in seed propagated crops but not in vegetatively propagated transgenic fruit trees. However, seed vigor becomes important when transgenic trees are used for breeding. It is well known that unintended effects in transgenic plants can be caused by changing environmental conditions or biotic and abiotic stressors [30], and seed quality can indicate these changes. Inheritance of transgenes in progeny has been investigated in earlier studies in apple [26,31] and plum trees [32,33]. Pear is another valuable fruit and is used in genetic engineering to transfer various valuable traits. Pear breeding is about as long as that of apple, and both genera have similarly long juvenile phases [34]. Hence it is important to shorten the breeding cycle of the fruit crop. We have studied the segregation of the *uidA* (β-glucuronidase) reporter gene in the progeny of field-grown transgenic pear trees for 7 years and the characteristics of the transgenic seeds.

## 2. Results

### 2.1. Inheritance of Transgenes in Progeny

**Figure 1:**
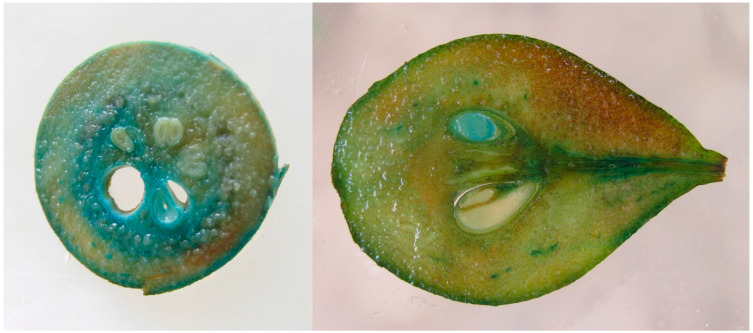
GUS staining of pear fruits: (**left**) transverse section; (**right**) longitudinal section.

The flowering of own-root GP217 pear rootstock trees was observed first in 2005—the eighth year after plantlets in vitro planting into a greenhouse and 5 years after planting in the field. They continued to produce flowers in the following years, and in 2007, 2009, 2010, 2011, and 2013, transgenic and control lines were pollinated with a mixture of pear pollen. Transgenic plants were indistinguishable visually from control in the morphology and color of flowers and the shape and size of fruits. Histochemical analysis showed *uidA* gene expression in fruit tissues, including seeds, with both GUS+ and GUS− seeds present within the same fruit (Figure 1).

**Figure 2:**
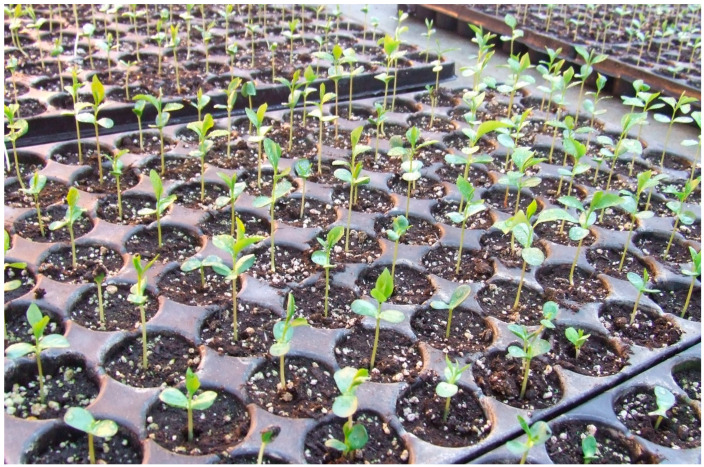
Pear seedlings in the greenhouse.

**Figure 3:**
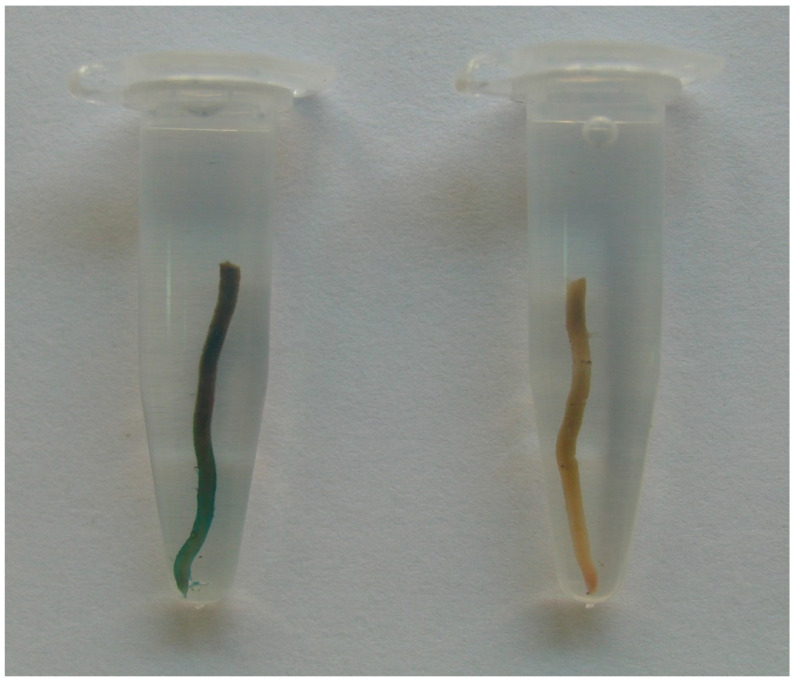
Histochemical GUS assay in roots of pear seedlings.

**Figure 4:**
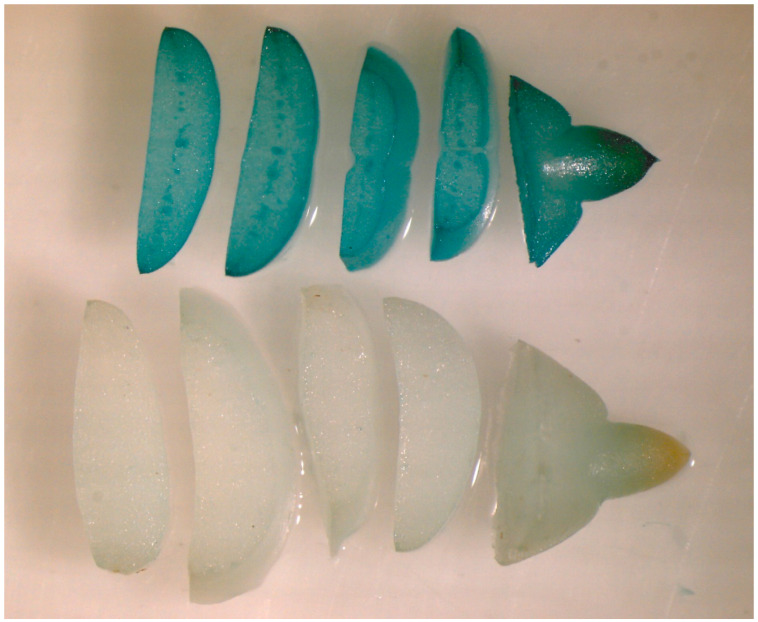
Histochemical GUS assay in pear seeds.

**Figure 5:**
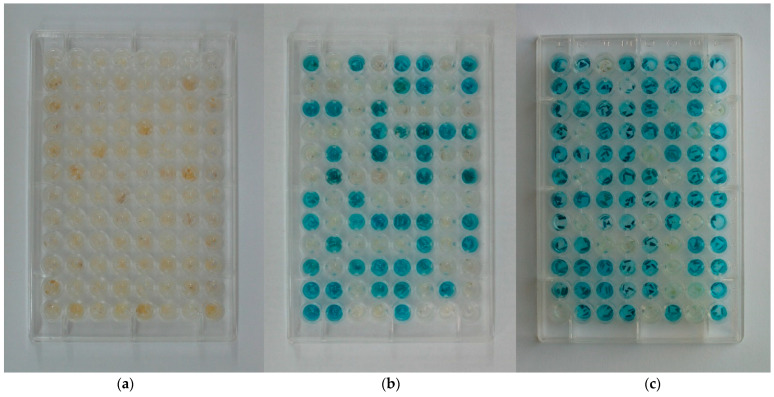
Segregation of GUS activity in pear progeny: (**a**) non-transgenic control; (**b**) ratio 1:1; (**c**) ratio 3:1.

The seeds collected from transgenic pear fruits looked normal, and we analyzed the inheritance of transgene in progeny. The *uidA* gene segregation in progeny was evaluated using GUS histochemical staining in two ways. Seeds of the 2007 and 2009 harvests were stratified and sown in a greenhouse to grow seedlings (Figure 2). GUS expression was evaluated in root samples (Figure 3). GUS staining was not observed in the roots of control plants, nor was it seen in the pear cultivars used for pollination. The evaluation procedure was time- and labor-consuming and the segregation in the harvests of 2010, 2011, and 2013 from the same plants was analyzed directly in seeds, peeled and cut into transverse segments. As with the root staining, we observed two clearly distinct variants: the presence or the absence of blue staining, without any intermediate variants (Figure 4). No staining was observed in seeds from control trees (Figure 5a), whereas seeds from transgenic lines showed a segregation ratio close to 1:1 or 3:1 (Figure 5b,c), which is consistent with Mendelian inheritance for one or two transgene loci, respectively.

**Figure 6:**
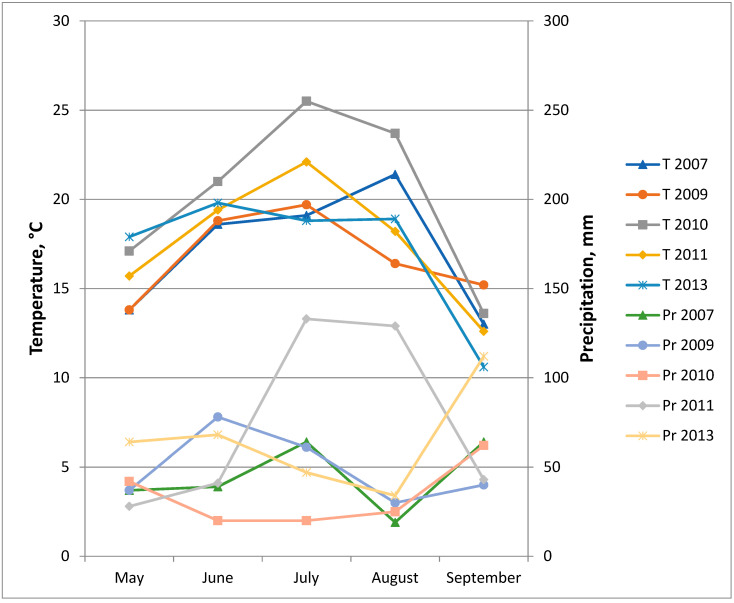
Weather conditions during growth seasons at field trial site (T—temperature, Pr—precipitation).

We evaluated seeds from five harvests obtained over 7 years (2007–2013). The weather conditions during the growth season (May–September) of those years are presented in Figure 6. The results of the *uidA* gene segregation analysis in pear progeny are summarized in Table 1. In total, we studied the progeny of 13 transgenic pear lines: 6 lines with *uidA*-int + *hpt* genes and 7 lines with *uidA* + *nptII* genes. As shown by the analysis using the Chi-square test, nine lines had a segregation ratio of 1:1, and four lines had a ratio of 3:1. Our data demonstrated the segregation stability over 5 harvests (7 years) in line NIII-2, four harvests (5 years) in lines HA-2 and NII-2, and three harvests (3–5 years) in seven lines. The 3:1 ratio was statistically confirmed in all cases, although it varied from 2.6:1 to 4:1. The 1:1 segregation ratio was not always statistically confirmed: there were three cases with a lack of GUS+ seeds (line NIII-5 in 2007, lines NII-2 and NIII-2 in 2011) and one case with an excess of GUS+ (line HA-2 in 2010). Those deviations from the 1:1 Mendelian ratio were apparently random because they were not reproduced in the subsequent years. Nor were they specific to any particular year, which could have indicated the influence of environmental conditions. For example, correct segregation was observed in seeds harvested in the abnormally hot and dry season of 2010 (Figure 6). The summary data for the whole analyzed period also statistically confirmed the 3:1 ratio for the four lines (Table 1). However, deviations were found for the 1:1 ratio: three lines lacked GUS+ samples, and one line had an excess.

### 2.2. Evaluation of Seed Characteristics

The physical parameters of seeds (weight and size) were also measured (Table 2 and Table 3). The non-transgenic seed weight varied from 26.8 to 30.9 mg over 7 years, i.e., it did not notably depend on environmental conditions. The extremely hot and dry weather in 2010 had little effect on seed weight, as did the heavy rainfall in July and August (fruit ripening period) in 2011. In most transgenic lines that had significant seed weight deviations from control, those deviations were random. However, in some lines (HA-3 and NIII-2), a significant decrease of seed weight was reproduced for several years. There was virtually no significant increase in seed weight compared to control. Seed size correlated with seed weight: a smaller weight was associated with smaller seed length and width. The most conservative feature was seed shape, i.e., the length to width ratio. This parameter varied very narrowly year-over-year (1.66–1.82), without any significant difference from the control.

Seed viability (germination) data are shown in Table 4. The germination rate in the harvest of 2007 ranged between 82.6 and 96.1% and was slightly lower—72.9 to 84.9%—in the harvest of 2009. In both harvests, the differences in germination rate among the pear genotypes were insignificant. We observed no difference in segregation patterns between viable seeds germinated after stratification (2007 and 2009) and seeds analyzed in a dormant state (2010, 2011, and 2013) (Table 1).

### 2.3. Stability of Transgene Expression in Progeny

The *uidA* gene expression was evaluated in randomly selected field-grown 3-year-old seedlings of the 2007 and 2008 harvests and one-year-old seedlings of the 2009 harvest grown in the greenhouse (3–4 plants per transgenic line). In general, there was no significant decrease of the expression levels in the progeny of the same line in the field compared to the greenhouse (Table 5). The progeny of about 40% of the lines showed similar expression levels (with up to a two-fold difference). The differences in the expression levels among the progeny of other lines were much greater, up to 20-fold. No cases of expression silencing were observed. Notably, the progeny of the four lines with two *uidA* gene loci had a more stable expression than those with one gene copy: three lines (HB-1, HA-4, NII-3) demonstrated two-fold differences, and only one line (NII-1) had a larger interval.

## 3. Discussion

The juvenile period in pear trees can reach 9–14 years for seedlings [35]. In this regard, pear breeding programs using both wild *Pyrus* species and different pear cultivars may require several generations of backcrosses and can continue for several decades [36]. To date, transgenic pear plants with resistance to herbicides [37], increased rooting ability [38], increased disease resistance [39], modified fruit taste [40], enhanced salt stress tolerance [41] have been obtained. These plants can be used for commercial cultivation and as donors of valuable traits. Fruiting of transgenic pear was first reported in 2008 [42], but there is still virtually no information on transgene inheritance in progeny. Most studies on transgene inheritance in other woody plants were carried out in the greenhouse, and just a few were done in the field, where transgenic trees performed as the female parents [16,26] or donors of transgenic pollen [27]. We evaluated transgene segregation in the progeny of many pear lines transformed with two binary plasmids and grown in the field for 7 years.

The studies showed stable inheritance of the *uidA* gene in the progeny of transgenic pear plants pollinated with non-transgenic pollen, with a segregation ratio of 1:1 (9 lines) or 3:1 (4 lines) corresponding to the Mendelian distribution for one and two loci, respectively. The inheritance of a transgene in tree progeny was first shown in Greensleeves apple trees with the *nos* and *nptII* marker genes, where the progeny of two lines showed segregation ratios of 1:1 and 3:1 [23]. Mendelian inheritance of other transgenes at a 1:1 ratio was later shown in apple [31] and plum [32,33]. Mendelian segregation in progeny was also noted in forest trees: poplar with the genes *Cry1Ac* and *nptII* demonstrated the ratios of 1:1 and 3:1 [16]. Other studies in trees, however, also showed non-Mendelian inheritance patterns. In the study of Yao et al. [2], one of five lines had a GUS+:GUS− seedling ratio that significantly differed from 1:1 (28% GUS+). The progeny of 2 out of 7 lines of field-grown Galaxy apple plants with an attacin E gene had a 1.7:1 or 2:1 segregation ratio, which was not consistent with the predicted 3:1 ratio [26]. A complex pattern of transgene inheritance was also noted in eucalyptus [10] and American chestnut [27]. The causes of non-Mendelian segregation were unknown [26] or attributed to tandem repeats [27].

The predicted segregation was not statistically confirmed in 4 of 38 samples of hybrid seeds evaluated in our study: there was a lack of GUS+ plants/seeds in 3 samples and their excess in one sample (Table 1). Although the presence of multiple gene copies is believed to increase the likelihood of non-standard segregation, all deviations observed in our study were in plants with a single transgene locus. As repeatedly reported earlier for crops, deviations from Mendelian inheritance were often manifested by a lack of transgenic plants rather than their excess [21]. The hybrid progeny of HoneySweet plum with PPV-CP gene pollinated in a greenhouse with pollen of two plum cultivars and *Prunus spinosa* also revealed a lack of transgenic plants: GUS+ were 49, 40, and 45% of the hybrids [32]. Such deviations could have been due to the insufficient size of the analyzed samples or preferential transgene transmission either through male or female gametes [21]. Histochemical staining showed stable inheritance of a *uidA* gene in the F1 progeny of *Betula platyphylla* through female or male gametes, but no segregation data were reported [43].

Excess of transgenic plants in the progeny is less common. Transgenic barley researchers attributed this to the small number of analyzed plants (sampling error) [44]. In general, the sample size is a very important factor: small size does not allow valid conclusions about segregation patterns. For example, 2 samples of as little as 7 and 19 hybrid seedlings of early-flowering apple obtained in [3], as well as samples of 4 to 15 seedlings of early-flowering pear obtained in [6,35] were too small for valid statistics. All these studies were carried out in a greenhouse, and Flachowsky et al. [3] believe that the small number of fruit and seed is the result of the stress induced by the greenhouse and the constitutive expression of the flower-inducing gene. We used at least a 50-seed sample per evaluation for valid statistical analysis. A study of segregation in T1-T3 generations of tobacco plants with the *nptII* gene also used 50–100 seeds per line [45]. Previously, researchers studied *uidA* gene inheritance in the progeny of fruit trees through histochemical staining of seedling leaves [2,32,33] but not seeds. We have demonstrated this method’s suitability for evaluating the segregation patterns in progeny. This method is faster, less labor-intensive, and can evaluate the segregation in all seeds, not only in the viable ones.

The segregation pattern in the pear progeny was stable over five harvests obtained in 7 years. There had been only one earlier report of repeated segregation analysis conducted in field-grown apple trees at a 6-year interval [26]. This analysis confirmed the persistence of Mendelian (two lines) and non-Mendelian (one line) inheritance patterns. Thus, environmental conditions did not cause deviations in inheritance patterns. It is known that changing environmental conditions can trigger unintended changes in transgenic plants [30], including the silencing of transgenes [15]. We also found no deviations in the segregation ratios despite fluctuations in growing conditions (Figure 6), particularly in the extremely hot and dry summer of 2010 [46]. Growing conditions, however, influenced the biochemistry of fruits: we observed a significant (2.5–3 times) increase of flavan content [47].

Despite the considerable number of evaluated lines (more than ever before), we did not find the ratio indicating three transgene loci (7:1), and there had not been any earlier reports of such ratio for trees. All studied woody plants were obtained by agrobacterial transformation, which, unlike bombardment, is characterized by a small number of copies with a prevalence of single ones [48]. The ratio of the number of pear lines with one/two loci was similar for both vectors: 4:2 for p35SGUS intron and 5:2 for pBI121. A similar ratio, 3:1, was also shown for poplar lines [16]. Using trees with a single transgene copy is preferable for breeding. For genes of valuable traits, it simplifies the inheritance pattern [26], and for early flowering genes, it increases the proportion of null segregants that do not contain transgenes [10].

One of the unintended effects of genetic engineering depending on inserted genes may be the alteration of plant fitness or reproductive success. It is known that the fitness of transgenic plants may react unexpectedly to stressful environmental conditions [30]. The fitness of a sexually propagating species is directly related to seed quality. Fitness deterioration in transgenic plants is more common than otherwise and is commercially undesirable. There are also known cases of improved fitness of transgenic plants [49,50], and this is a biosafety concern. The biosafety regulatory requirements are one of the reasons for the limited commercial use of GM fruit trees [51]. Many studies have shown a positive correlation between seed weight and germination and/or the subsequent growth of seedlings in various tree species [52,53,54]. As far as we know, the seed quality of transgenic trees has not been evaluated before. To assess the seed quality of the pear, we used physical (weight and size of seeds) and physiological (germination) methods. Most of the transgenic pear lines did not show a stable change in seed weight. The transgenic progeny’s seed shape (length:width) did not change either. Stable grain shape and the absence of changes in grain weight and germination in rice with a drought resistance gene *CaMsrB2* grown in the field for two years under irrigation or drought provided the grounds to conclude that the rice was suitable for commercial cultivation [55].

In our study, the hot and dry weather in 2010 did not affect the quality of pear seeds, although high-temperature stress in the field significantly reduced the pea seed weight (by 8–15%) and germination rate [56]. The difference in grain weight between superior and inferior grains might affect germination vigor [57]. We found no significant differences in the germination of transgenic seed compared to control, although such differences had been reported earlier for horticultural plants. For unknown reasons, the germination rate was low in kiwi hybrids with *nptII* and *uidA* genes: the seeds of most hybrids did not germinate at all [58]. The very slow and low germination rate (15% germination after several months) was observed for T1 seeds of the cultivated strawberry (*Fragaria* × *ananassa* Duch.) with *nos* and *nptII* marker genes [59]. The germination rates of early flowering apple plants from various crosses varied from 44 to 75% [25]. The absence of any differences in segregation between the germinated and dormant seeds suggests that the seed viability did not affect the inheritance patterns in the progeny of the transgenic pear.

The expression of the *uidA* gene in the generative progeny of the pear was stable: no silencing was detected in any seedling at the age of 3 years [60]. It is known that unintended changes often occur when transgenic plants are transferred from controlled greenhouse conditions to more variable field conditions [61]. Therefore, it is important to evaluate the phenotypic differences between transgenic lines and their non-transgenic analogs in field trials [62]. Nor did we observe any significant decrease in the expression levels in progeny grown in the greenhouse or in the field (Table 4). However, exposure to stress affected the levels of expression: most plants in the abnormally hot year of 2010 had a lower *uidA* gene expression than in 2011. A significantly increased (2.5–3 fold) flavan content in pear fruits in 2010 can indicate the experienced stress from high temperatures [47].

The progeny of pear lines with two loci had a more stable transgene expression, with less variable expression levels (Table 4). Contradictory data were obtained on the variation of gene expression levels in the progeny from one cross. Flachowsky et al. [3] evaluated resistance to *Erwinia amylovora* of apple hybrids between transgenic early-flowering line and the fire blight-resistant wild species *Malus fusca*. In nine tested F1 apple seedlings, the expression stability varied from high, like the donor for fire blight resistance, to low, comparable to susceptible control cultivar. Ma et al. [43] reported varying expression of the *Bgt* gene across hybrid progeny of birch. On the other hand, the resistance against *Clostera anachoreta* and *Hyphantria cunea* of the progeny of insect-resistant poplar was similar to that of the parent line and had no abrupt fluctuations [16]. The relative transgene expression in transgenic offspring of American chestnut (two T1 individuals from two lines) was approximately similar to the parent and differed from each other by about 2–2.5 times [27].

In conclusion, our results have demonstrated that transgene inheritance in the generative progeny of field-grown pear trees follows Mendelian segregation and does not depend on changing environmental conditions. The transgene expression in progeny is stable, and transgenic seeds’ physical and physiological characteristics do not change. These findings allow the use of transgenic pear trees in breeding programs.

## 4. Materials and Methods

### 4.1. Plant and Seed Material

The transgenic GP217 pear clonal rootstock (*Pyrus communis* L.) plants were used in this study. These plants were produced by *Agrobacterium*-mediated transformation using the binary plasmid pBI121 (35S-*uidA* and nos-*nptII* genes) or the p35SGUSintron (35S-*uidA* gene with an intron and nos-*hpt* gene) [63]. Non-transgenic GP217 was used as the control wild type in all experiments. Transgenic and control plants were planted to the field in spring 2000 in accordance with the permission of the Russian Inter-Agency Committee on Genetic Engineering Activity (authorization # 48-P/00) [42]. Flowers were hand-pollinated using a fresh pollen mix of three diploid pear cultivars (Alenushka, Belorusskaya Pozdnyaya, Eseninskaya) in 2007–2011 and 2013. Fruits were harvested at the maturity stage (August), seeds were removed from the fruits and dried at room temperature. Seed weight and size parameters was determined in four replicates of 25 or 50 seeds, depending on the available amount. The length and width of the seeds were measured with a caliper, and the shape was calculated as the ratio of length to width. Temperature and precipitation data were collected from the nearest weather station (8 km from the field trial site).

### 4.2. Transgene Segregation in Progeny

The segregation of the *uidA* gene in pear progeny was determined by histochemical analysis of GUS expression in two ways. The seeds of 2007 and 2009 were stratified at 4 °C for three months to break dormancy and were sown in a mixture of peat and perlite (3:1) in the greenhouse in spring. Root samples were collected from seedlings and assayed for *GUS* expression in microtubes. The seeds of 2010, 2011, and 2013 were cleaned from the seed coat, then they were cut into transverse segments and were placed into the wells of a 96-well microtiter plate. Histochemical staining of GUS activity was performed by overnight incubation of the roots or seeds in an *X-Gluc* solution at 37 °C [64].

### 4.3. Expression of uidA Gene in Progeny

Three (harvest 2007 and 2009) of four (harvest 2008) plants were randomly selected from GUS-positive progeny of transgenic lines for quantitative assessment of GUS activity [60]. The plants were grown in a greenhouse for one season and planted in the field in autumn. Expression was determined in three-year-old plants in the field (harvest 2007 and 2008 in 2010 and 2011, respectively) and one-year-old plants in the greenhouse (harvest in 2009 in 2010). Fluorometric assay of leaf tissue was conducted as described by Scott et al. [64] using MUG as substrate and the Infinite 200 multifunctional microplate reader (Tecan Group Ltd., Männedorf, Switzerland) at Pushchino Center for Collective Use of Science Equipment. Protein concentration in the plant extracts was measured according to the Bradford method [65], and GUS activity was expressed as pmol 4-MU/min per mg protein.

### 4.4. Statistical Analysis

Data are expressed as mean ±SE. Differences between averages were analyzed by ANOVA using the Dunnett’s test to compare transgenic versus control groups (STATISTICA 10). A Chi-square test was used to analyze the deviation of observed segregation ratios in progeny from the expected Mendelian ratios. Chi-squares with less than 0.05 probability were considered significant.

## Figures and Tables

**Table 1:** Segregation of *uidA* genes in progeny of transgenic pear.

Transgene	Lines	Ratio	2007		2009		2010		2011		2013		Total	
		Analyzed	GUS^+/−^	χ^2^	GUS^+/−^	χ^2^	GUS^+/−^	χ^2^	GUS^+/−^	χ^2^	GUS^+/−^	χ^2^	GUS^+/−^	χ^2^
control	GP217		0/123		0/109		0/127		0/106		0/92		0/557	
*uidA*-int	HB-1	3:1	nd		78/25	0.029	nd		101/39	0.610	38/13	0.007	217/77	0.222
	HA-2	1:1	nd		nd		nd		nd		22/33	2.200	22/33	2.200
	HA-3	1:1	nd		69/72	0.064	39/23	4.129 *	84/66	2.160	70/63	0.368	262/224	2.971
	HA-4	1:1	75/53	3.781	nd		nd		nd		44/48	0.174	119/101	1.473
	HA-5	3:1	83/26	0.076	nd		nd		nd		nd		83/26	0.076
	HA-6	1:1	114/91	2.580	58/44	1.922	104/89	1.166	nd		nd		276/224	5.408 *
*uidA*	NII-1	3:1	148/51	0.042	88/27	0.142	147/45	0.250	nd		nd		383/123	0.129
	NII-2	1:1	nd		46/51	0.258	88/104	1.333	44/76	8.533 **	26/29	0.164	204/260	6.759 *
	NII-3	3:1	nd		73/28	0.399	100/25	1.667	111/38	0.020	nd		284/91	0.108
	NIII-2	1:1	24/25	0.020	39/28	1.806	42/54	1.500	29/48	4.688 *	38/50	1.636	172/205	2.889
	NIII-4	1:1	25/27	0.077	83/71	0.935	73/85	0.911	nd		nd		181/183	0.011
	NIII-5	1:1	59/92	7.212 **	19/28	1.723	24/23	0.021	nd		nd		102/143	8.126 **
	NIV-2	1:1	76/95	2.111	69/93	3.556	70/81	0.801	nd		nd		215/269	6.025 *

χ^2^—the critical value is 3.84 (*p* < 0.05); *, **—significant deviation from expected ratio at *p* = 0.05 or 0.01; nd—not determined.

**Table 2:** Seed weight of pear progeny (mg).

Line	2007	2009	2010	2011	2013
GP217	30.9	29.3	26.8	30.5	27.5
HB-1	nd	27.6	nd	31.2	22.4 ***
HA-2	nd	nd	nd	nd	27.2
HA-3	nd	22.7 ***	24.1	26.5 **	19.6 ***
HA-4	30.3	nd	nd	nd	28.2
HA-5	29.9	nd	nd	nd	nd
HA-6	31.2	29.4	28.1	31.3	nd
NII-1	30.9	28.4	27.9	30.0	nd
NII-2	nd	30.1	28.8	32.8	30.0 *
NII-3	nd	30.3	25.1	32.5	nd
NIII-2	29.3	18.1 ***	22.4 **	30.2	23.5 ***
NIII-4	29.1	22.1 ***	27.0	29.6	nd
NIII-5	27.1 ***	22.4 ***	26.8	nd	nd
NIV-2	30.8	19.6 ***	19.6 ***	31.1	nd

Data are expressed as mean ±SE (*n* = 4). *, **, ***—significance at *p* < 0.05, 0.01, and 0.001, respectively (Dunnett’s test).

**Table 3:** Size (mm) and shape (length:width) of pear seeds.

Line	2007			2009			2010			2011			2013		
	Length	Width	Shape	Length	Width	Shape	Length	Width	Shape	Length	Width	Shape	Length	Width	Shape
GP217	8.3	4.9	1.68	8.5	5.0	1.73	8.3	4.9	1.72	8.4	4.9	1.72	8.3	4.8	1.72
H-1	nd	nd	nd	8.4	5.0	1.69	nd	nd	nd	8.3	4.9	1.72	8.0	4.8	1.69
H-2	nd	nd	nd	nd	nd	nd	nd	nd	nd	nd	nd	nd	8.3	4.9	1.69
H-3	nd	nd	nd	8.4	4.8	1.75	8.2	4.7	1.74	8.1 *	4.6 ***	1.76	7.9	4.5 *	1.76
H-4	8.1	4.7 *	1.72	nd	nd	nd	nd	nd	nd	nd	nd	nd	8.3	4.8	1.74
H-5	8.2	4.7	1.72	nd	nd	nd	nd	nd	nd	nd	nd	nd	nd	nd	nd
H-6	8.4	5.0	1.69	8.4	4.8	1.76	8.3	4.9	1.72	8.3	5.0	1.67	nd	nd	nd
NII-1	8.2	5.0	1.66	8.4	4.9	1.72	8.6	5.1	1.69	8.3	5.0	1.68	nd	nd	nd
NII-2	nd	nd	nd	8.3 *	4.5 ***	1.82 *	8.7	4.9	1.75	8.5	5.0	1.70	8.2	4.9	1.69
NII-3	nd	nd	nd	8.5	4.9	1.74	8.2	4.8	1.71	8.3	4.9	1.71	nd	nd	nd
NIII-2	8.2	4.8	1.72	8.3 *	4.8	1.74	8.0	4.6 *	1.73	8.4	4.9	1.73	8.0	4.7	1.71
NIII-4	8.4	4.9	1.71	8.3	4.8	1.74	8.4	4.9	1.72	8.5	4.8	1.77	nd	nd	nd
NIII-5	7.7 ***	4.7 *	1.66	8.3	4.7 *	1.75	8.3	4.9	1.72	nd	nd	nd	nd	nd	nd
NIV-2	8.1	4.9	1.67	8.2 *	4.6 ***	1.79	7.8 **	4.5 **	1.74	8.3	4.9	1.71	nd	nd	nd

Data are expressed as mean ± SE (*n* = 4). *, **, ***—significance at *p* < 0.05, 0.01, and 0.001, respectively (Dunnett’s test).

**Table 4:** Germination of transgenic pear seeds.

Line	Germination Rate, %	
	2007	2009
GP217	88.6 ± 3.0	76.6 ± 7.0
HB-1	-	75.2 ± 4.2
HA-2	-	84.9 ± 3.7
HA-3	89.1 ± 3.6	-
HA-4	82.9 ± 1.5	-
HA-6	89.7 ± 5.3	72.9 ± 5.4
NII-1	93.6 ± 3.2	74.2 ± 4.6
NII-2	-	76.4 ± 5.3
NII-3	-	81.5 ± 4.3
NIII-2	86.0 ± 3.1	77.9 ± 6.9
NIII-4	85.3 ± 2.8	73.0 ± 4.0
NIII-5	88.0 ± 4.6	78.3 ± 7.3
NIV-2	96.3 ± 1.1	73.6 ± 4.0

**Table 5:** Expression of *uidA* genes in the transgenic progeny of pear.

Gene	Line	Locus	GUS Activity—Average (min–max), pmol 4-MU/min/µg Protein		
		Number	Crossing 2007 (Field 2010)	Crossing 2008 (Field 2011)	Crossing 2009 (Greenhouse 2010)
*uidA*-int	HB-1	2	nd	72.8 (50.1–100.4)	11.6 (10.5–12.6)
	HA-2	1	nd	nd	2.5 (0.6–5.1)
	HA-3	1	10.4 (1.0–16.8)	nd	nd
	HA-4	2	8.4 (6.9–10.7)	38.9 (27.6–47.4)	nd
	HA-5	1	nd	18.0 (11.9–29.6)	nd
	HA-6	1	10.5 (1.7–36.0)	nd	18.3 (7.6–35.4)
*uidA*	NII-1	2	5.2 (1.3–14.9)	35.1 (30.5–41.0)	14.5 (7.3–25.7)
	NII-2	1	nd	nd	5.0 (3.8–7.2)
	NII-3	2	nd	23.0 (13.5–30.6)	3.7 (2.4–5.3)
	NIII-2	1	4.9 (1.4–6.7)	nd	2.5 (1.3–4.7)
	NIII-4	1	9.5 (1.4–21.2)	nd	5.0 (1.7–11.2)
	NIII-5	1	3.0 (0.7–7.9)	7.3 (1.1–19.4)	nd
	NIV-2	1	2.2 (1.3–3.2)	nd	8.2 (1.1–19.2)

## Data Availability

Not applicable.
